# Research on fault-tolerant decision algorithm for data security automation

**DOI:** 10.3389/fdata.2025.1600540

**Published:** 2025-10-20

**Authors:** Jianxin Li, Ruchun Jia, Ning Xiang, Yizhun Tian

**Affiliations:** ^1^China School of Cyberspace Security, Changzhou College of Information Technology, Changzhou, Jiangsu, China; ^2^College of Computer Science, Sichuan University, Chengdu, Sichuan, China; ^3^School of Artificial Intelligence, Leshan Vocational and Technical College, Leshan, Sichuan, China; ^4^School of Computer Science, Civil Aviation Flight University of China, Guanghan, Sichuan, China

**Keywords:** data source security, multi angle analysis, automation, fault tolerance, operation and maintenance decision, niche mechanism

## Abstract

**Introduction:**

Traditional operation and maintenance decision algorithms often ignore the analysis of data source security, making them highly susceptible to noise, time-consuming in execution, and lacking in rationality.

**Methods:**

In this study, we design an automated operation and maintenance decision algorithm based on data source security analysis. A multi-angle learning algorithm is adopted to establish a noise data model, introduce relaxation variables, and compare sharing factors with noise data characteristics to determine whether the data source is secure. Taking the ideal power shortage and minimum maintenance cost as the objective function, we construct a classical particle swarm optimization model and derive the expressions for particle search velocity and position. To address the problem of local optima, a niche mechanism is incorporated: the obtained automated data is treated as the population, a reasonable number of iterations is determined, individual fitness is stored, and the optimal state is obtained through a continuous iterative update strategy.

**Results:**

Experimental results show that the proposed strategy can shorten operation and maintenance time, enhance the rationality of decision-making, improve algorithm convergence, and avoid falling into local optima.

**Discussion:**

In addition, fault-tolerant analysis is performed on data source security, effectively eliminating bad data, preventing interference from malicious data, and further improving convergence performance.

## 1 Introduction

Power stations in China are large in scale, dispersed in different regions, and equipped with numerous power equipment. Therefore, how to realize rational operation and maintenance of the power system has become a prominent problem. With the development of the power grid information integration platform, information services will be fully promoted. However, due to the large number of businesses and users, combined with the emergence of new technologies such as the Internet of Things, automated operation and maintenance decision-making has become a pressing issue. At the same time, a large number of power information collection devices are connected to the power grid, generating massive volumes of power grid big data. However, some bad or false data may exist in the collected data due to malicious attacks or sensor failures. If these data are used directly in operation and maintenance decision-making, they can affect the assessment of the real state of the power grid, impact decision results, and even cause serious economic losses.

Therefore, to improve scientific decision-making, Xue et al. (2019) proposed a data-driven optimization algorithm for operation and maintenance of the power acquisition system. They analyzed the structure of the power data acquisition system and constructed an operation and maintenance scheme based on the historical database collected by the system. According to the operation and maintenance cycle of different regions and the safety requirements of the power grid, an optimization operation and maintenance model is established, and the particle swarm optimization algorithm is used to solve the model. Zhang et al. (2020) designed a power data operation analysis algorithm. The algorithm includes grid-related geographic information, service information, and distribution information. Through an improved machine learning method to analyze the correlation and risk level of power grid data, it is beneficial for the operation and maintenance decisions of the power sector. Although the above two algorithms can extract false information in power grid big data, they ignore the analysis of data source security, which leads to the algorithm being greatly affected by noise, and the application process taking a long time and being of poor rationality. In order to further improve the data security analysis, domestic and foreign experts in related fields have also put forward some good research results. Suryaprabha and Saravana Kumar (2020) take a wireless sensor network as the research environment, because a wireless sensor network can detect various types of information from the environment and deliver it to users in real time. Information is stored in the cloud and accessible to users. However, because information is part of collaborative communication and therefore vulnerable to attack, node-level building algorithms are not secure, which provides the scope for this study to focus on algorithms that can be used for denial-of-service attacks, as they minimize network performance.

In recent years, research on operational optimization and data source security has advanced considerably, yet important gaps remain. For instance, while Por et al. (2024) provide a comprehensive taxonomy of attacks and countermeasures in graphical passwords, and Gabr et al. (2024) demonstrate biologically inspired approaches to secure data modeling, these findings have not yet been systematically integrated into fault-tolerant optimization for complex infrastructures. At the same time, swarm intelligence has emerged as a promising paradigm in critical infrastructures, with Khan et al. (2025) presenting an adaptive model for distributed grid security that strengthens the theoretical foundation of swarm-based optimization methods. Moreover, Adebimpe et al. (2023) highlight recognition-based authentication strategies resistant to noise and shoulder-surfing, offering insights that can enhance anomaly detection and robust AI-driven decision-making in data source validation. In this study, scholars proposed an optimized energy-based constrained DoS (Denial of Service) detection algorithm, namely the Optimized energy-Based constrained Scheme (OBES) algorithm, used to deal with denial-of-service attacks, which learns network traffic and manages intruders to realize security analysis of wireless sensor network data sources. Wang et al. (2020) proposed that the current communication network cannot handle large-scale malicious code attacks, and the network security mechanism is weak, resulting in a serious lag in response. Therefore, they proposed a security response method for a multi-service communication network under malicious code attacks. Based on the timeliness and destructiveness of malicious code, the K-L divergence value of a Gaussian mixture distribution is obtained, and the optimal divergence solution is obtained by using an approximate calculation. The minimum distance clustering center is used to complete the malicious code clustering. The component mean is re-estimated by the maximum expectation algorithm, the cumulative value of the mean is analyzed by weighting, and the eigenvalues are extracted. Based on the risk minimization criterion of malicious code attack, the optimal classification surface function of the least square support vector machine is transformed into an optimization problem. By introducing the Lagrange operator, the equality constraint and Lagrange constraint conditions are replaced. The kernel function is created according to the quadratic programming problem solution obtained, so as to achieve the derivation of the decision function of the support vector machine. After the normalization of the security response risk level and the optimization of kernel parameters and regularization parameters, the data source security is analyzed. However, the above two data security analysis methods ignore the extraction of noisy data features, resulting in more noise in the process of data analysis, leading to a certain deviation in data security analysis results.

Based on the above research results and background content, this study combines the multi-angle learning (Hussain, 2017) method with the niche particle swarm method to realize automatic operation and maintenance decisions. Multi-angle learning is a kind of machine learning (Hawezi et al., 2022), and observations from different perspectives are usually complementary. If the data from multiple perspectives are placed in the same subspace, the shared factors existing in all perspectives can be obtained through continuous mining, and then the data source information can be traced, and the security of the data source can be judged to provide a necessary data security guarantee for operation and maintenance decisions. Through the improvement of the particle swarm optimization algorithm and the use of niche sharing theory, the diversity of population distribution can be improved to avoid local optimal decision results, which can be better applied in power grid operation and maintenance problems.

## 2 Multi-angle data source security analysis

Big data plays a key role in the stable operation of the power grid, but with the continuous expansion of the network scale, it also faces great difficulties, the most important of which is the challenge of data quality. Accurate estimation of power system state relies on good data security. Inaccurate or missing data will interfere with the decision-making analysis of dispatchers and even cause wrong judgments, which will bring huge losses to people's production and life.

### 2.1 Data source security analysis problem modeling

If it represents the observation data vectors collected by the operation and maintenance system in a certain period of time, these vectors form a matrix in a certain time series. Assuming that they are observed through various perspectives, a multi-angle sample set *X* = {*X*^(1)^, *X*^(2)^, ..., *X*^(*V*)^} is generated.

If a dictionary is used to represent the data source from any perspective, then write:


(1) $$\begin{array}{l} X^{\left(V\right)}=A^{\left(V\right)}Z^{\left(V\right)}+E^{\left(V\right)} \end{array}$$

In the formula, *Z*^(*V*)^ represents the coefficient matrix, and *E*^(*V*)^ describes the noise information. Because there will be shared information in multi-angle information, the shared factors are placed together in a certain subspace. At this time, *Z*^(*V*)^ should belong to the block matrix with low-rank features (Kong et al., 2017; Zhang and Zhou, 2019). Since *Z*^(*V*)^ describes shared factors, different *Z*^(*V*)^ will have certain similarities. In addition, both false data and attack data are bursty, so these data are not included in the scope of the sharing factor (Zhang, 2021; Collins et al., 2017).

Divide the observation data into two parts: sharing factor and noise data, and find out false data and attack data from the noise data. For the number of observations, the number of these unsafe data is limited, so sparsity will be reflected in the data matrix. The noise data model is constructed by the *L*_1_ norm, and the following objective equation is obtained (Li et al., 2019):


(2) $$\begin{array}{l} \begin{array}{c} \underset{Z^{\left(V\right)},E^{\left(V\right)}}{\min}=\overset{V}{\underset{V=1}{\sum }}\left(rank\left(Z^{\left(V\right)}\right)+\alpha ||E^{\left(V\right)}|{|}_{1}\right)\\ +\beta \overset{V}{\underset{V=1}{\sum }}\overset{V}{\underset{j=V+1}{\sum }}||Z^{\left(V\right)}-Z^{\left(j\right)}|{|}_{F}^{2}\\ s.tX^{\left(V\right)}=A^{\left(V\right)}Z^{\left(V\right)}+E^{\left(V\right)},v=1,2,\ldots ,V \end{array} \end{array}$$

In the formula, ||·||_1_ represents the *L*_1_ norm, ||·||_*F*_ is the Frobenius norm, and both are balance parameters. In Formula 2, before the plus sign, it can be used for sparse detection of test data (Chen et al., 2020; Zhao et al., 2020), and after the plus sign, it can reduce the similarity of shared factors (Wu et al., 2020; Chen et al., 2019). Because it is unknown in the equation, it is initialized to make it become, then the Formula 2 is transformed into the following equivalent problem:


(3) $$\begin{array}{l} \begin{array}{c} \underset{Z^{\left(V\right)},E^{\left(V\right)}}{\min}=\overset{V}{\underset{V=1}{\sum }}\left(||Z^{\left(V\right)}|{|}_{*}+\alpha ||E^{\left(V\right)}|{|}_{1}\right)\\ +\beta \overset{V}{\underset{V=1}{\sum }}\overset{V}{\underset{j=V+1}{\sum }}||Z^{\left(V\right)}-Z^{\left(j\right)}|{|}_{F}\\ s.tX^{\left(V\right)}=A^{\left(V\right)}Z^{\left(V\right)}+E^{\left(V\right)},v=1,2,\ldots ,V \end{array} \end{array}$$

In the formula, ${\left||Z^{\left(V\right)}|\right|}_{*}$ is the nuclear norm of *Z*^(*V*)^.

### 2.2 Dangerous data source detection

This article only analyzes data source security issues from two perspectives, that is, 11. Due to the low-rank constraints in 22, slack variables are added, and the optimized equivalent form of Formula 3 at 22 is obtained:


(4) $$\begin{array}{l} \begin{array}{c} \underset{Z^{\left(1\right)},E^{\left(1\right)},Z^{\left(2\right)},E\left(2\right)}{\min}=\overset{2}{\underset{V=1}{\sum }}\left(||J^{V}|{|}_{*}+\alpha ||E^{\left(v\right)}|{|}_{1}\right)+\beta ||Z^{\left(1\right)}-Z^{\left(2\right)}|{|}_{F}^{2}\\ s.tX^{\left(V\right)}=A^{\left(V\right)}Z^{\left(V\right)}+E^{\left(V\right)},Z^{\left(V\right)}=J^{\left(V\right)},V=1,2 \end{array} \end{array}$$

In the formula, *J*^(*V*)^ represents the optimization variable, and the Formula 4 is solved by the Lagrangian method, and the following expression is obtained:


(5) $$\begin{array}{l} \sum_{V=1}^{2}\left\|J^{\left(V\right)}\right\|=\langle Y_{1}^{\left(V\right)},X^{\left(V\right)}-X^{\left(V\right)}Z^{\left(V\right)}-E^{\left(V\right)}\rangle \\ \;+2/\mu ({\left\|Y_{2}^{\left(V\right)}X^{\left(V\right)}-X^{\left(V\right)}Z^{\left(V\right)}-E^{\left(V\right)}\right\|}_{F}^{2}\\ \;+{\left\|Z^{\left(V\right)}-J^{V}\right\|}_{F}^{2}) \end{array}$$

In the above formula, $Y_{1}^{\left(V\right)}$ and $Y_{2}^{\left(V\right)}$ are the data source Lagrangian multipliers under the two perspectives, μ belongs to the balance parameter (Peng et al., 2019; Ding et al., 2020). Treat the quantity other than *Z*^(1)^ as a constant, calculate its partial derivative, and set the partial derivative equal to 0, then the solution to obtain *Z*^(1)^ is expressed as:


(6) $$\begin{array}{l} Z^{\left(1\right)}=\left(X^{\left(1\right)}X^{\left(1\right)}\right)\left(\begin{array}{c} X^{\left(1\right)}\left(X^{\left(1\right)}-E^{\left(1\right)}\right)+J^{\left(1\right)}\text{+}\\ Z^{\left(2\right)}+\frac{X^{\left(1\right)}Y_{1}^{\left(1\right)}-Y_{2}^{\left(1\right)}+\beta Z^{\left(2\right)}}{\mu } \end{array}\right) \end{array}$$

In the same way, the solution of *Z*^(2)^ is obtained. And solve *E*^*V*^ by the following formula:


(7) $$\begin{array}{l} E^{\left(V\right)}=\\ \underset{E^{\left(V\right)}}{\arg\min}\overset{2}{\underset{V=1}{\sum }}\left(\begin{array}{c} \alpha /\mu ||E^{\left(V\right)}|{|}_{2,1}+\\ 1/2||E^{\left(V\right)}-\left(X^{\left(V\right)}-X^{\left(V\right)}Z^{\left(V\right)}+Y_{1}^{\left(V\right)}/\mu \right)|{|}_{F}^{2} \end{array}\right) \end{array}$$

Use Formulas 6, 7 to continuously optimize and iterate, and judge the acceptance conditions after each iteration. If convergence occurs, stop updating; otherwise, continue to iterate until the conditions are met. If the data source of the *i* observed value is safe, the difference *Z*^(*V*)^ will be very similar, and the product of the difference *Z*^(*V*)^ is larger at this time (Zhang and Ma, 2020). By obtaining the value of $Z_{i}^{\left(V\right)}$ and $E_{i}^{\left(V\right)}$, it can be judged whether the data source is safe.

Using the above method to determine whether the data source collected by the power grid is safe, remove false and attack data, and use valid data for operation and maintenance decision-making will greatly improve the rationality of decision-making.

## 3 Operation and maintenance decision-making with an improved ant colony algorithm

### 3.1 Objective function determination

The operation and maintenance system mainly provides services for the following projects:

Scheduling: It involves using layered thinking to conduct integrated management of power dispatching and is responsible for the command and management of the regional information system. The operation and maintenance target it faces is the entire power system and all staff related to dispatching.

Maintenance: Periodic maintenance and emergency repair are important components of maintenance. In addition, it is necessary to ensure effective equipment maintenance and acceptance (Tang and Zhou, 2020).

Combined with the main items of operation and maintenance, the objective function of operation and maintenance decision-making is set. Usually, equipment maintenance will not cut off the load directly, but it will increase the risk of a power outage. Therefore, this study regards the ideal power shortage as the objective function.

The ideal power shortage: the ideal power shortage is the sum of the power loss caused by the shutdown of the power grid during the maintenance period, which can indicate the degree of power grid reliability reduction caused by emergency repairs (Zhou and Long, 2020; Zhang et al., 2019).


(8) $$\begin{array}{l} \min f_{1}=\overset{T}{\underset{t=1}{\sum }}\left(\underset{x\in S_{i}}{\sum }C_{x}\overset{M}{\underset{i=1}{\prod }}P_{i}^{x_{i}}{\left(1-P_{i}\right)}^{1-x_{i}}\right)T_{t} \end{array}$$

In the formula, *T* represents the number of maintenance periods, *S*_*t*_ represents the collection of load-shedding states in the *t* time period during the maintenance process, *x* = (*x*_1_, ..., *x*_*n*_) State vector describing the element, *C*_*x*_ represents the load shedding amount of the fault state *x*, *M* is the number of components, $x_{i^{\prime }}$ Describe the operating state of the component *i*′ at this time, $P_{i^{\prime }}$ represents the probability that the component will fail, *T*_*t*_ is the number of hours for the overhaul process.

Overhaul cost: Overhaul cost represents the total economic cost of the equipment during the overhaul process. The cost of different maintenance methods is quite different; for example, the cost of different maintenance times is also different.


(9) $$\begin{array}{l} \min f_{2}=\overset{N}{\underset{i}{\sum }}\overset{T}{\underset{i=1}{\sum }}p_{i}^{t}z_{i}^{t}u_{i}^{t} \end{array}$$

In the formula, *N* represents the total number of equipment that needs to be overhauled, $p_{i^{\prime \prime }}^{t}$ is the time cost when a working group overhauls the line *i*″, including overtime pay for holiday maintenance, $z_{i^{\prime \prime }}^{t}$ represents the team that overhauls the line *t* within *i*″ time periods, $u_{i^{\prime \prime }}^{t}$ is the maintenance status of the equipment. These include $u_{i^{\prime \prime }}^{t}$ and $u_{i^{\prime \prime }}^{t}$.

### 3.2 Automated operation and maintenance aid decision making

#### 3.2.1 Classical particle swarm model

In the classic particle swarm optimization method, the solution generated by the optimization problem is regarded as a particle in the search space, and after multiple iterations, the optimal solution is obtained (Zhao et al., 2019). During the iterative process, the particles track the best position *P*_*best*_ and the global best position *G*_*best*_, and constantly update their own positions. The speed and position of the particle search are given by the following formula:


(10) $$\begin{array}{l} u_{i^{*}d}^{t+1}=wu_{i^{*}d}^{t}+c_{1}r_{1}\left(p_{i^{*}d}^{t}-y_{i^{*}d}^{t}\right)+c_{2}r_{2}\left(p_{gd}^{t}-y_{i^{*}d}^{t}\right) \end{array}$$

(11) $$\begin{array}{l} y_{i^{*}d}^{t+1}=x_{i^{*}d}^{t}+u_{i^{*}d}^{t+1} \end{array}$$

In the formula, *t*′ is the number of iterations, *w* is the weight information (Liu E. G. et al., 2019; Yang et al., 2019), *c*_1_ and *c*_2_ are the acceleration constants of the two iterations (Lu et al., 2021), *r*_1_and *r*_2_ are random numbers between [0, 1], *u*_i*d_ and $y_{i^{*}d}$ are, respectively the *d* velocity and position components of particle i^*^, describes the optimal position of particle i^*^. Also known as *P*_*best*_, *p*_*gd*_ is the global optimal position of the particle i^*^. That is *G*_*best*_.

#### 3.2.2 The decision-making process of the niche particle swarm optimization algorithm

When optimizing for multiple objectives, the global best position is not just one, but has multiple undominated best positions (Zhou et al., 2020). Therefore, this study transforms individual particle swarms into multi-objective particle swarms and proposes a niche particle swarm method.

(1) Individual and global best position determination
  For the individual optimal position, if the particle termination is effective, the particle will dominate *P*_*best*_, then take *P*_*best*_ as the current position; If it has a dominant effect on the current position of *P*_*best*_ particles, in this case *P*_*best*_ will remain unchanged (Jin and Yang, 2020). If there is no dominance relationship between the two, just choose one of them arbitrarily. The global optimum is determined by combining the fitness values of all solutions in the external archive.
(2) External archive
  Use the external archive method to store the best solution obtained in the iterative process, and organize the external files to improve the calculation efficiency. If the number of particles in the file is higher than the specified number, the minimum fitness particles are removed to ensure that all solutions in the file are evenly distributed (Tang et al., 2020).
Niche sharing mechanism
  Express the fitness of an individual $Y_{i^{*}}$ in the file as:

(12) $$\begin{array}{l} F_{i^{*}}=\frac{1}{S_{i^{*}}},i^{*}=1,2,...,N_{S} \end{array}$$

In the formula, *N*_*s*_ represents the number of individuals in the niche, and $S_{i^{*}}$ describes the sharing degree of an individual $Y_{i^{*}}$. The expression is as follows:


(13) $$\begin{array}{l} S_{i^{*}}=\overset{N_{S}}{\underset{j=1}{\sum }}f_{Sh}^{\prime }\left(d_{i^{*}j}\right),j=1,2,\ldots ,N_{S} \end{array}$$

$f_{sh}^{'}\left(d_{i^{*}j^{\prime }}\right)$ in the formula is the sharing function of individuals $X_{i^{*}}$ and $X_{j^{\prime }}$, it reflects the correlation between two individuals in the niche (Liu X. J. et al., 2019).

When there are more individuals, the greater the degree of sharing, the smaller the fitness; on the contrary, the higher the fitness value. Niche sharing is to use this method to reduce individual adaptability, and then minimize the occurrence of local optimum and premature phenomena in the iterative process (Tan et al., 2020; Liu Y. D. et al., 2019).

The process of making automated operation and maintenance decisions through the above methods is as follows:

Step 1: Initialize the population, generate the original position and velocity, and set a reasonable number of iterations.
Step 2: Determine the objective functions of all particles, and place the solutions without a dominance relationship in the external file.
Step 3: Calculate the fitness of all individuals in the file, compare these values, and select an individual as the global optimal position.
Step 4: Constantly update the position and velocity of the incoming particles through Formulas 11, 12, and update accordingly.
Step 5: Update the file through the solution without a dominance relationship.
Step 6: If the number of individuals in the external file exceeds the set maximum value, the individual with the lowest fitness should be removed at this time.
Step 7: If the end requirements are met, no longer search, and output the best operation and maintenance strategy from the external file; otherwise, go back to step 3 and continue (Zhang, 2020).

## 4 Simulation experiment analysis

The simulation experiment takes a power grid as the research object to verify the effectiveness of the proposed automatic operation and maintenance decision-making algorithm based on the security analysis of data sources. The hardware environment for simulation experiments utilizes an Intel Core i7-12700K processor (3.6 GHz, 12 cores, 20 threads), equipped with 32 GB DDR4-3200 memory, a 1TB NVMe SSD, and an NVIDIA GeForce RTX 3060 graphics card (12 GB VRAM). The software environment was based on the Windows 10 Professional (64-bit) operating system, utilizing MATLAB R2022b as the simulation platform. Code was written in the MATLAB scripting language, with data processing performed using Python 3.9 (equipped with the Pandas 1.5.3 and NumPy 1.24.3 libraries). The experimental dataset is based on actual operation and maintenance data from a regional power grid during January to March 2023. It comprises 1,000 samples, each containing timestamps, power plant ID, substation ID, load values, data acquisition perspective, and data labels. This dataset is used to validate the experimental process and assess algorithm performance. The experimental parameter settings are shown in Table 1.

**Table 1:** Parameter settings.

**Parameter name**	**Value**
Balance parameter λ1	0.1
Balance parameter λ2	0.05
Number of iterations max_iter	100
Convergence threshold ε	1e-5
Population size *N*	50
Maximum number of iterations T	70
External archive capacity Q	30
Inertia weight w	0.5~0.9 (linearly decreasing)
Acceleration constants c1, c2	2.0, 2.0
Sharing radius σ	0.8
Ideal power shortage weight α	0.6
Maintenance cost weight β	0.4

**Figure 1:**
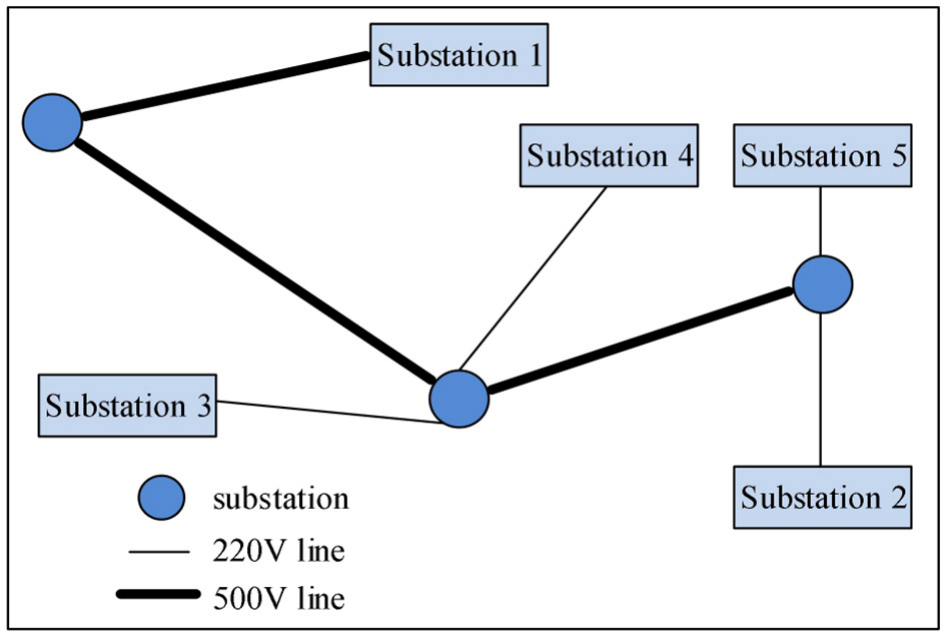
Schematic diagram of the power grid architecture in a certain place.

Assume that the regional power grid consists of five power stations and several substations. When a fault occurs and a large-scale power outage occurs, the method in this study, the data-driven algorithm, and the power data analysis system are used to give different recovery plans for the power stations. The power grid structure of the region is shown in Figure 1.

The fault recovery sequence of the three algorithms is simulated using the grid architecture. The three algorithms give the fault recovery sequence as follows:

The algorithm in this study: power plant 3 - power plant 2 - power plant 5 - power plant 4 - power plant 1;
Literature (Xue et al., 2019) algorithm: power plant 1 - power plant 3 - power plant 4 - power plant 5 - power plant 2;
Literature (Zhang et al., 2020) algorithm: power plant 2 - power plant 5 - power plant 4 - power plant 3 - power plant 1.

**Figure 2:**
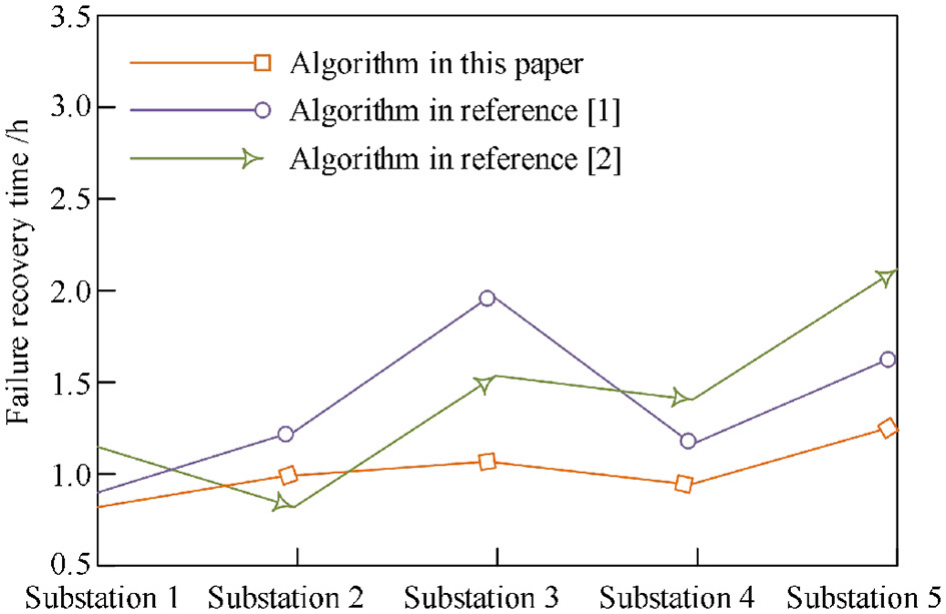
Comparison chart of fault repair time of different algorithms.

According to three different sequences for emergency repair, the required recovery time is shown in Figure 2. According to the experimental results, it can be seen that the fault repair sequence in this study has an obvious advantage in time. For different substations, the fault recovery time of this method is up to 1.2 h, and the shortest is 1.0 h. Although for substation 2 and substation 3, the emergency repair time of this method is not the shortest, but it takes the least time overall, and all emergency repair tasks can be completed in the shortest time. In contrast, the failure recovery time of the method in Kong et al. (2017) is the longest at 2.0 h and the shortest at 1.3 h. The longest fault recovery time of the method in Zhang and Zhou (2019) is 1.5 h, and the shortest is 0.8 h.

The main reason why the proposed method has this application advantage is that the proposed method takes the minimum power shortage as the objective function, and at the same time, makes the most accurate decision based on the collected safety data.

**Figure 3:**
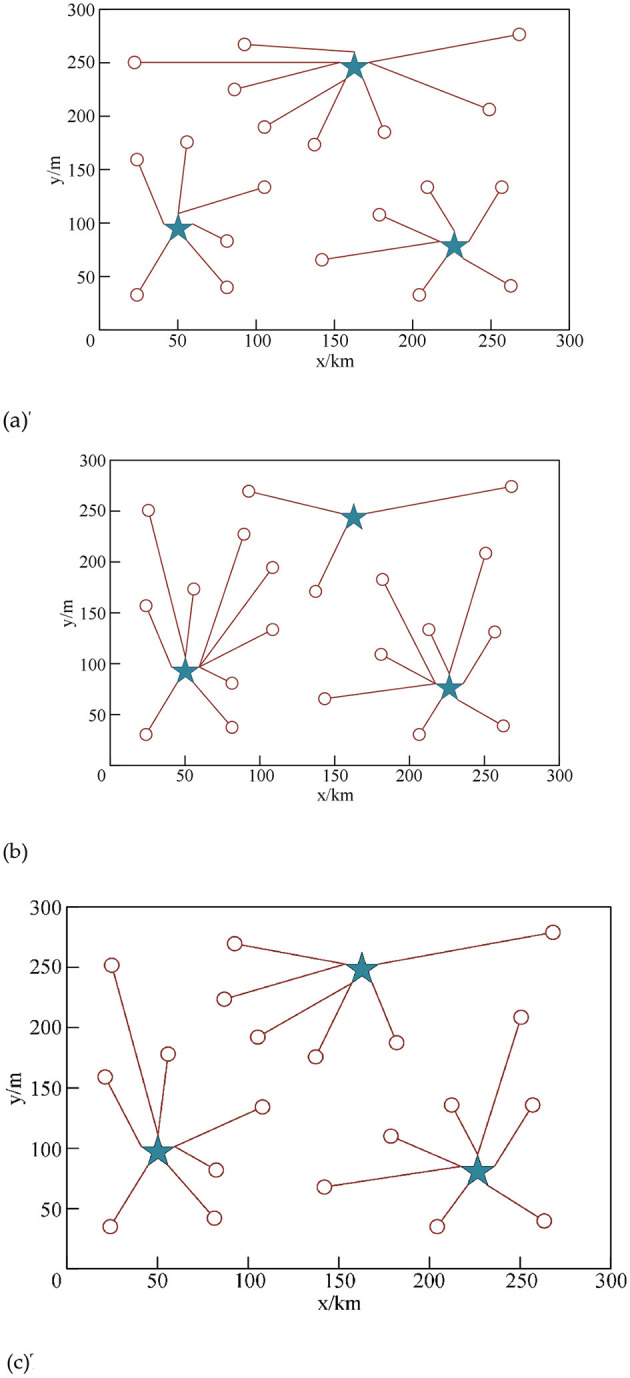
Allocation results of the algorithm in different algorithms. **(a)** The results of the area assignment of the algorithm in this study. **(b)** The results of the area assignment of the algorithm in literature (Xue et al., 2019). **(c)** The results of the area assignment of the algorithm in literature (Zhang et al., 2020).

In order to verify the rationality of the allocation of emergency repair points by the algorithm in this study, it is known that there are three emergency repair stations in this area, and the overall area is divided into 20 small areas. The above three algorithms are used to match the emergency repair points in these small areas, and the obtained configuration results are shown in Figures 3a–c, respectively. The star in the figure represents the emergency repair station, and the circle represents the small area, and the connection between them is the allocation of the area to the connected emergency repair station.

From the above experimental results, it can be seen that the proposed algorithm can reasonably allocate the sub-areas corresponding to the emergency repair station according to the distance; in the algorithm of literature (Kong et al., 2017), the emergency repair station (175, 250) only allocates three areas, and there is a large difference in the number of allocations; while the result given by the algorithm in literature (Zhang and Zhou, 2019) guarantees the equalization of the workload of the emergency repair station, but it cannot be allocated according to the constraint of the shortest distance. In contrast, the decision result given by the method in this study is the best. The experimental results show that using the proposed algorithm to complete automatic operation and maintenance can greatly reduce the operation and maintenance cost.

Based on the above experimental results, in order to further prove the convergence performance of the algorithm, the number of iterations is set to 70, and the optimal solution for each generation of the optimal decision result is obtained by comparing the algorithm in the literature (Kong et al., 2017), the algorithm in the literature (Zhang and Zhou, 2019), and the algorithm proposed in this study. attribute value.

**Figure 4:**
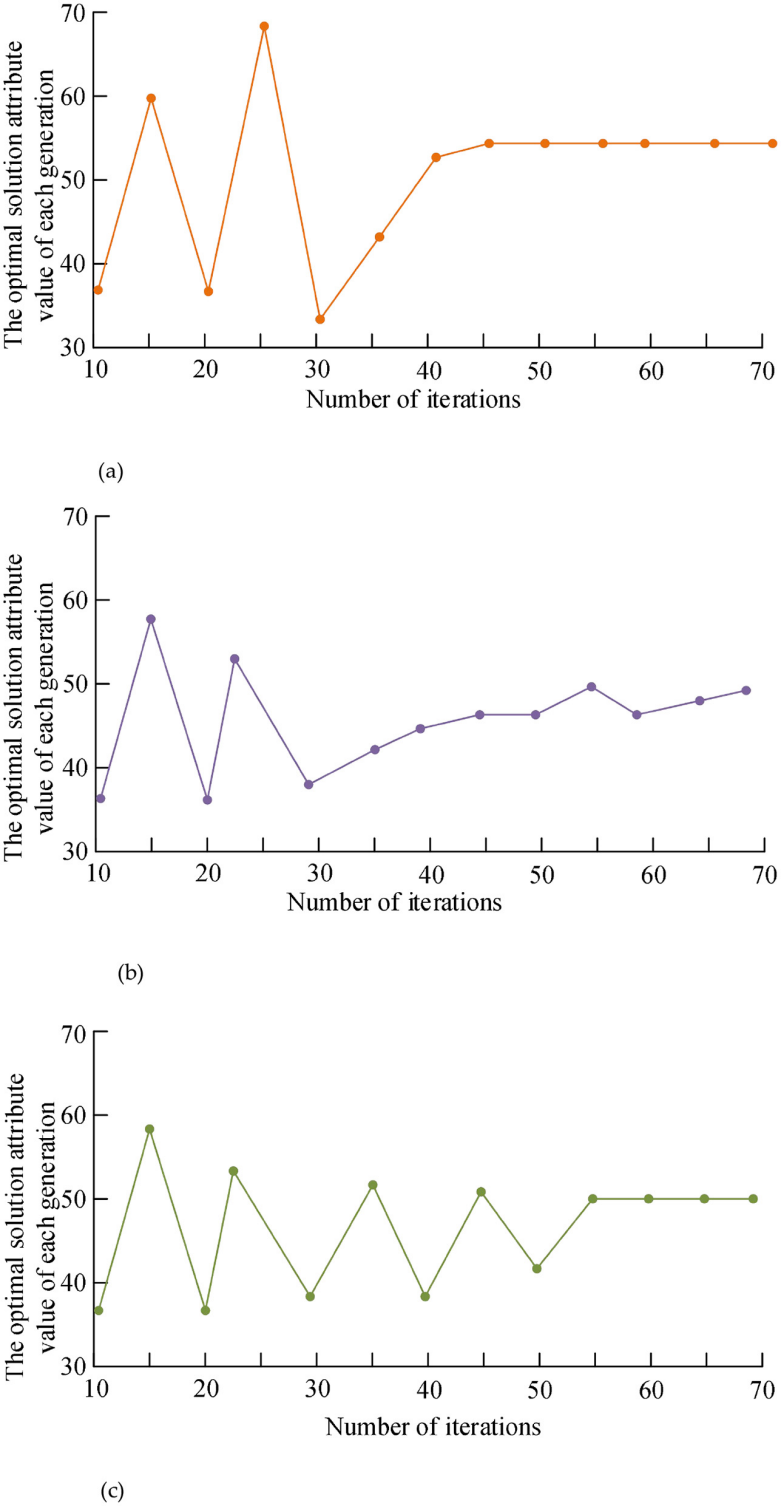
Convergence performance comparison of different algorithms. **(a)** Convergence performance of the algorithm in this study. **(b)** Convergence performance of literature (Kong et al., 2017) algorithm. **(c)** Convergence performance of the literature (Zhang and Zhou, 2019) algorithm.

It can be seen from Figures 4a–c that although the algorithm in literature (Kong et al., 2017) can present a stable trend within the shortest number of iterations, there will still be certain fluctuations in the stable process; the convergence time of the algorithm in literature (Zhang and Zhou, 2019) is long; Some improvements have been made to determine the best individual position and population position, improve the convergence performance of the algorithm, and avoid falling into local optimum. On the other hand, this study analyzes the security of data sources, which can effectively remove bad data, ensure that the algorithm will not be disturbed by malicious data, and improve the convergence performance.

To clarify the independent contributions of the three core components of multi-angle analysis, noise filtering, and niche Particle Swarm Optimization (PSO) to algorithm performance, four sets of ablation experiments were designed, namely: Experimental group A (complete algorithm): the algorithm proposed in this study, which includes multi angle analysis, noise filtering, and niche PSO; Experimental group B (removing multi-angle analysis): only retaining noise filtering and niche PSO, using single perspective data observation in the data source security analysis stage; Experimental group C (removing noise filtering): only retaining multi-angle analysis and niche PSO, without constructing a noise data model (Formulas 2–7), and directly using the original collected data for decision-making; Experimental Group D (Removing Small Habitat PSO): Only multi-angle analysis and noise filtering are retained, and the classical particle swarm optimization algorithm (without small habitat sharing mechanism and external archives) is used to solve the objective function. The experimental indicators were selected as the total duration of fault recovery, decision rationality (average distance of emergency repair point allocation), and algorithm convergence iteration times. Each experiment was repeated 10 times, and the average value was taken. The results are shown in Table 2.

**Table 2:** Results of the ablation experiment.

**Experimental group**	**Total time for fault recovery/h**	**Average distance of repair point allocation/km**	**Convergence iteration times**
A (complete algorithm)	5.8 ± 0.2	3.2 ± 0.3	42 ± 3
B (remove multi-angle analysis)	7.5 ± 0.4	4.8 ± 0.5	45 ± 4
C (remove noise filtering)	8.2 ± 0.5	5.3 ± 0.6	58 ± 5
D (remove small habitat PSO)	6.9 ± 0.3	3.5 ± 0.4	65 ± 6

According to Table 2, experimental group A (complete algorithm) performs the best in all three indicators, with a total fault recovery time of 5.8hree i an average distance of 3.2hree indicators, lts are shown in Table esfaul and a convergence iteration of 42dicators, Compared with experimental group A, experimental group B(excluding multi-angle analysis)increased the total time for fault recovery to 7.5taltia, the average distance for emergency repair point allocation to 4.8esfault and the number of convergence iterations slightly increased to 45esfault r Experimental group C (removing noise filtering)showed the most significant deterioration in various indicators, with a total fault recovery time of 8.2ecover an average distance of 5.3y time of 8.2cators, showed the mos and an increase in convergence iterations to 58howed the The total time for fault recovery in experimental group D (excluding small habitat PSO) was 6.9owed t with an average distance of 3.5bitat PSO was 6.9owed the most si5 The number of convergence iterations increased significantly to 65sfault re In summary, multi perspective analysis can improve the accuracy of data security judgments by mining shared factors from multiple perspectives, laying the foundation for rational emergency repair planning; Noise filtering can eliminate false and attack data, reduce iterative interference to ensure accurate decision-making and search efficiency; Small habitat PSO can prevent algorithms from falling into local optima, significantly improve convergence speed, and achieve optimal algorithm performance through the synergistic effect of the three. All of them are necessary components for the efficient and reliable operation of the automated operation and maintenance decision-making algorithm in this study.

## 5 Conclusion

The automated operation and maintenance system can effectively improve the low efficiency of decision-making, but there is also the risk of bad data attacks. In this study, based on the analysis of data source security, the particle swarm optimization algorithm is improved to enhance convergence performance. Simulation experiments show that this method can reasonably provide operation and maintenance decision-making results, ensure grid stability to the greatest extent, and improve economic benefits. At the same time, with the support of this decision-making method, the efficiency of troubleshooting, daily maintenance, and other tasks has been greatly improved, thereby enhancing user satisfaction and enterprise competitiveness. Nevertheless, several limitations should be acknowledged. While our chosen parameters proved effective in this study, their optimality across all potential scenarios cannot be guaranteed; automating parameter tuning represents a valuable direction for future research to enhance adaptability and ease of use. Moreover, although the present study demonstrates the overall efficacy of the proposed integrated framework, a thorough ablation study to isolate the contribution of each component (e.g., the niche mechanism, multi-angle preprocessing) would provide deeper insights. Beyond this, broader benchmarking against recent hybrid optimization and anomaly detection algorithms, a formal convergence and stability analysis, and more extensive testing on larger, noisier, and more complex power systems will be essential to more convincingly validate the robustness, scalability, and practical applicability of the approach.

## Data Availability

Publicly available datasets were analyzed in this study. This data can be found here: jiaruchun@stu.scu.edu.cn.

## References

[B1] Adebimpe L. A. Ng I. O. Idris M. Y. I. Okmi M. Ang T. F. Por L. Y.. (2023). Systemic literature review of recognition-based authentication method resistivity to shoulder-surfing attacks. Appl. Sci. 13:10040. 10.3390/app131810040

[B2] Chen H. Y. Qi H. W. Yin H. (2019). Integrated solution for intelligent operation and maintenance of cloud data center in the field of aerospace test mission. Microelectr. Comput. 36, 33–37. 10.19304/j.cnki.issn1000-7180.2019.05.008

[B3] Chen W. H. Tian Z. Zhang L. Z.. (2020). Outlier detection method for high-dimensional sparse data based on interpolation. Comput. Eng. Sci. 42, 966–972. 10.3969/j.issn.1007-130X.2020.06.003

[B4] Collins S. Deane J. P. Poncelet K. Panos E. Pietzcker R. C. Delarue E.. (2017). Integrating short term variations of the power system into integrated energy system models: a methodological review. Renew. Sust. Energy Rev. 76, 839–856. 10.1016/j.rser.2017.03.090

[B5] Ding Y. Wang Y. J. Tian L. Wang B. Y. Yuan F. Zhang K. (2020). Smart grid data security aggregation scheme supporting third-party arbitration. Acta Electr. Sin. 48, 350–358. 10.3969/j.issn.0372-2112.2020.02.018

[B6] Gabr M. Diab A. Elshoush H. T. Chen Y. L. Por L. Y. Ku C. S.. (2024). Data security utilizing a memristive coupled neural network in 3D models. IEEE Access 12, 116457–116477. 10.1109/ACCESS.2024.3447075

[B7] Hawezi R. S. Khoshaba F. S. Kareem S. W. (2022). A comparison of automated classification techniques for image processing in video internet of things. Comput. Electr. Eng. 101:108074. 10.1016/j.compeleceng.2022.108074

[B8] Hussain M. I. (2017). Internet of Things: challenges and research opportunities. CSI Trans. ICT 5, 87–95. 10.1007/s40012-016-0136-6

[B9] Jin B. Yang P. (2020). Analysis of archive data security management strategy in the era of big data. Inform. Sci. 38, 30–35. 10.13833/j.issn.1007-7634.2020.09.005

[B10] Khan A. A. Yang J. Laghari A. A. Baqasah A. M. Alroobaea R. Ku C. S.. (2025). BAIoT-EMS: consortium network for small-medium enterprises management system with blockchain and augmented intelligence of things. Eng. Appl. Artif. Intell. 141:109838. 10.1016/j.engappai.2024.109838

[B11] Kong Y. Kong Z. Liu Z. Wei C. Zhang J. An G. (2017). Pumped storage power stations in China: the past, the present, and the future. Ren. Sust. Energy Rev. 71, 720–731. 10.1016/j.rser.2016.12.100

[B12] Li Y. Q. Li D. D. Wang Z. Zhang J. (2019). Multi-view learning with gravitational nearest neighbor classifier. Comput. Eng. Applic. 55, 137–142.+179. 10.3778/j.issn.1002-8331.1805-0448

[B13] Liu E. G. Wang L. Yi C. J.. (2019). Secure data transmission of substation terminal based on aggregate signature. Comput. Eng. Design 40, 1809–1815. 10.16208/j.issn1000-7024.2019.07.002

[B14] Liu X. J. Ye W. Jiang J. W. Zhang L. (2019). Data security storage scheme in hybrid cloud mode. J. Beijing Univ. Technol. 45, 295–303. 10.15918/j.tbit1001-0645.2019.03.01230149142

[B15] Liu Y. D. Wang X. Tu G. S. Wang H. (2019). Full life cycle cloud outsourcing data security audit protocol. Comput. Applic. 39, 1954–1958. 10.11772/j.issn.1001-9081.2018122438

[B16] Lu J. D. Xiao R. Z. Jin S. Y. (2021). Research progress of cloud data security. J. Electr. Inform. 43:11. Available online at: https://jeit.ac.cn/cn/article/pdf/preview/10.11999/JEIT200158

[B17] Peng Z. Zhang X. Zhang Q. Su D. Huo X. (2019). Remote operation and maintenance technology of substation monitoring system based on GSP. Power Autom. Equip. 39, 210–216. 10.16081/j.issn.1006-6047.2019.04.031

[B18] Por L. Y. Ng I. O. Yang J. Chen Y. L. Ku C. S. (2024). A systematic literature review on the security attacks and countermeasures used in graphical passwords. IEEE Access 12, 53408–53423. 10.1109/ACCESS.2024.3373662

[B19] Suryaprabha E. Saravana Kumar N. M. (2020). Retracted article: enhancement of security using optimized DoS (denial-of-service) detection algorithm for wireless sensor network. Soft Comput. 24, 10681–10691. 10.1007/s00500-019-04573-4

[B20] Tan P. L. Wang X. J. Tang W. Q. Wan L. X. R. (2020). Design of MCPs data security architecture based on blockchain. Comput. Eng. Design 41:7. 10.16208/j.issn1000-7024.2020.12.00731915982

[B21] Tang X. Shan W. Liu D. Zhou L. (2020). Cloud data security de duplication method against side channel attack based on threshold re encryption. J. Commun. 41:14. 10.11959/j.issn.1000-436x.2020103

[B22] Tang X. Zhou L. N. (2020). Cloud data security de duplication method against additional block attack based on response fuzziness. Comput. Applic. 40:6. 10.11772/j.issn.1001-9081.2019081468

[B23] Wang X. Y. Liu Q. J. Pang G. L. (2020). Security response simulation of multi service communication network under malicious code attack. Comput. Simul. 37, 137–141. 10.3969/j.issn.1006-9348.2020.10.030

[B24] Wu W. L. Liu G. H. Zhang J. B. (2020). Complexity analysis of function query solution on big data. Comput. Appli. 40:4. 10.11772/j.issn.1001-9081.201909161825435347

[B25] Xue L. Zheng T. Guo L. Ren W. Huang L. H. Zeng X. J. (2019). Research on optimal operation and maintenance of power user power acquisition system based on data drive. Electr. Measur. Instrument. 56, 42–46. 10.19753/j.issn1001-1390.2019.017.008

[B26] Yang G. Ding H. Zou J. Jiang H. Chen Y. (2019). Big data security scheme based on high-performance password. Comput. Res. Dev. 56:2207. 10.7544/ISSN1000-1239.2019.20190390

[B27] Zhang J. (2021). Distributed network security framework of energy internet based on internet of things. Sust. Energy Technol. Assess. 44:101051. 10.1016/j.seta.2021.101051

[B28] Zhang L. (2020). Research on storage data security of network service cloud platform based on OSI model. Modern Electr. Technol. 43:5. 10.16652/j.issn.1004-373x.2020.05.017

[B29] Zhang M. Zhou Z. P. (2019). Deep subspace clustering with low rank constraint a priori. Pattern Recognit. Artif. Intell. 32, 652–660. 10.16451/j.cnki.issn1003-6059.201907009

[B30] Zhang S. G. Xian H. Q. Liu H. Y. Wang L. M. (2019). Cloud encrypted data security deduplication method. J. Softw. 30, 3815–3828. 10.13328/j.cnki.jos.005610

[B31] Zhang T. Ma H. Q. (2020). Collaborative research on open data and data security policy based on policy text calculation. Inform. Theory Pract. 43:8. 10.16353/j.cnki.1000-7490.2020.06.023

[B32] Zhang W. Sheng W. Du S. (2020). Architecture and technical implementation of distribution network operation analysis system based on massive data. Power Syst. Autom. 44, 147–153. Available online at: https://link.cnki.net/urlid/32.1180.TP.20191205.1203.002

[B33] Zhao H. X. Li Y. Shi H. B. (2020). Research on Weighted Naive Bayesian algorithm based on high-dimensional data. Stat. Decis. Ma. 36, 5–9. 10.13546/j.cnki.tjyjc.2020.08.001

[B34] Zhao Z. Wang J. Zhu Z. Sun L. (2019). Attribute based encryption scheme for secure sharing of Internet of things data. Comput. Res. Dev. 56, 1290–1301. 10.7544/issn1000-1239.2019.20180288

[B35] Zhou W. K. Long M. (2020). Secure transmission scheme of environmental monitoring data based on blockchain. Comput. Sci. 47, 315–320. 10.11896/jsjkx.190100195

[B36] Zhou X. X. Liu W. G. Sui H. M. Cheng H. Y. (2020). Five safes security framework and its enlightenment to the security access of sensitive data in China's library field. Inform. Theory Pract. 43:6. 10.16353/j.cnki.1000-7490.2020.03.015

